# Electronic Health Record Use Patterns Among Well-Being Survey Responders and Nonresponders: Longitudinal Observational Study

**DOI:** 10.2196/64722

**Published:** 2025-02-04

**Authors:** Daniel Tawfik, Tait D Shanafelt, Mohsen Bayati, Jochen Profit

**Affiliations:** 1 Department of Pediatrics, Stanford University School of Medicine, Palo Alto, CA, United States; 2 Department of Medicine, Stanford University School of Medicine, Stanford, CA, United States; 3 Stanford Medicine WellMD & WellPhD Center, Stanford, CA, United States; 4 Department of Operations, Information and Technology, Stanford Graduate School of Business, Stanford, CA, United States

**Keywords:** electronic health record metadata, electronic health record, EHR, electronic medical record, patient record, health records, personal health record, use, response bias, well-being, burnout, physicians, experiences, surveys, longitudinal studies, observational studies

## Abstract

**Background:**

Physician surveys provide indispensable insights into physician experience, but the question of whether responders are representative can limit confidence in conclusions. Ubiquitously collected electronic health record (EHR) use data may improve understanding of the experiences of survey nonresponders in relation to responders, providing clues regarding their well-being.

**Objective:**

The aim of the study was to identify EHR use measures corresponding with physician survey responses and examine methods to estimate population-level survey results among physicians.

**Methods:**

This longitudinal observational study was conducted from 2019 through 2020 among academic and community primary care physicians. We quantified EHR use using vendor-derived and investigator-derived measures, quantified burnout symptoms using emotional exhaustion and interpersonal disengagement subscales of the Stanford Professional Fulfillment Index, and used an ensemble of response propensity-weighted penalized linear regressions to develop a burnout symptom prediction model.

**Results:**

Among 697 surveys from 477 physicians with a response rate of 80.5% (697/866), always responders were similar to nonresponders in gender (204/340, 60% vs 38/66, 58% women; *P*=.78) and age (median 50, IQR 40‐60 years vs median 50, IQR 37.5‐57.5 years; *P*=.88) but with higher clinical workload (median 121.5, IQR 58.5‐184 vs median 34.5, IQR 0‐115 appointments; *P*<.001), efficiency (median 5.2, IQR 4.0-6.2 vs median 4.3, IQR 0‐5.6; *P*<.001), and proficiency (median 7.0, IQR 5.4‐8.5 vs median 3.1, IQR 0‐6.3; *P*<.001). Survey response status prediction showed an out-of-sample area under the receiver operating characteristics curve of 0.88 (95% CI 0.77-0.91). Burnout symptom prediction showed an out-of-sample area under the receiver operating characteristics curve of 0.63 (95% CI 0.57-0.70). The predicted burnout prevalence among nonresponders was 52%, higher than the observed prevalence of 28% among responders, resulting in an estimated population burnout prevalence of 31%.

**Conclusions:**

EHR use measures showed limited utility for predicting burnout symptoms but allowed discrimination between responders and nonresponders. These measures may enable qualitative interpretations of the effects of nonresponders and may inform survey response maximization efforts.

## Introduction

Symptoms of burnout among physicians have risen sharply in recent years [1], but burnout symptoms and other markers of physician well-being are currently identified by voluntary responses to surveys [2]. Such intermittent surveys often have low response rates and carry the risk of response bias, as physicians with burnout or other symptoms of poor well-being may have systematically different likelihood of responding [3]. Although prior analyses have shown few significant differences between early and late responders or between initial responders and those who respond to incentivized secondary surveys of nonresponders [1,4], in many studies, information about nonresponders is limited to standard demographics. Evaluating survey results in relation to EHR use measures reflecting the work environment and experiences of each physician may allow a more detailed analysis of the differences between responders and nonresponders, thereby enabling extrapolations relevant to the well-being of nonresponders.

Symptoms of burnout affect primary care physicians disproportionately [1,4,5] and pose a threat to quality of care and patient safety [6,7]. Almost 50% of primary care physicians believe that the amount of time spent on clerical tasks is unreasonable [8], those with a high electronic health record (EHR) task load have a higher likelihood of burnout [9-11], and up to 75% believe that the EHR contributes to burnout [12,13]. Although these findings may suggest that EHR-based measures could be used to predict burnout symptoms, the performances of EHR-based measures in prediction models have been limited [14,15]. This combination of findings suggests that a more complex interplay exists among EHR use, physician well-being, and survey response.

This study sought (1) to identify systematic differences in EHR use measures between physician survey responders and nonresponders and (2) to provide a prediction modeling approach for estimating symptom burden among survey nonresponders using physician burnout as a use case.

## Methods

### Overview and Study Design

We used a repeated cross-sectional observational design to assess a wide range of EHR use characteristics in relation to burnout symptoms. EHR measures were compiled by the institution’s EHR vendor (Epic Systems) or calculated from the local EHR data repository. Burnout measures relevant to this study were part of larger routine surveys on professional fulfillment at Stanford University School of Medicine.

### Ethical Considerations

This study was approved by the institutional review board at Stanford University School of Medicine (IRB-49374) with deidentification processes enabling the waiver of informed consent. Data were deidentified prior to analysis. Incentives previously provided for survey completion are detailed below, but no further compensation was provided for inclusion in this study.

### Study Participants

All primary care physicians at a large US academic medical center and its community affiliates were surveyed as part of routine quality improvement efforts and professional fulfillment benchmarking. Participants were invited via email, followed by 4 reminder email requests over the following 6 weeks at 2 time points (2019 and 2020). Participation was voluntary, with local department chairs, division chiefs, and well-being champions allowed to encourage participation at their discretion. At the academic medical center, a department-level financial incentive of US $50 per respondent was offered to the 3 departments with the highest response rates. At the community affiliate, survey completion was a prerequisite for individuals to receive their annual clinical bonuses. The primary stated purpose of the survey was to “help inform our efforts to make operational improvements to make Stanford University a better place for all to practice, educate, and conduct research.” The survey was administered by a third party (SullivanLuallin Group), and all responses were deidentified to preserve confidentiality.

Each physician was categorized as a “never responder” if they did not provide survey responses at either time point, “always responder” if they responded to each yearly invitation received, or “partial responder” if they were invited both years but only responded once.

### Burnout Measures

Surveys were distributed at the academic medical center in May 2019 and October 2020 and distributed at the community affiliate in March 2019 and March 2020. We used the Stanford Professional Fulfillment Index, which includes 10 prompts assessing burnout symptoms, 4 related to work exhaustion and 6 related to interpersonal disengagement. Details of this index and full burnout prompts are provided in prior publications [16,17]. In line with prior research [17], we calculated the mean score for these 10 burnout items, which are scored 0‐4 on a Likert scale, then transposed to a 0‐10 scale by multiplying by 2.5. Although all analyses were conducted on continuous scores, for ease of reporting, we dichotomized such that a score of ≥3.325 was considered indicative of burnout symptoms, in line with the validation results for this instrument [17].

### EHR Use Measures

For each physician, we compiled EHR use measures for the 3-month period leading up to survey administration. We used Signal data compiled by Epic Systems to approximate key measures of workload, use, and efficiency, as defined in Epic’s UserWeb. We calculated additional measures of workload from the institution’s EHR data repository, including number of unique patients with appointments, number of patients with multiple appointments in a given month, sentiment scores of progress notes (range −1 to +1 for maximally negative to maximally positive sentiment), and principal components. We reduced this large feature set of 1677 overlapping predictors to a smaller set of 214 candidate predictors by manually curating to measures with low collinearity, high cardinality, and known or potential associations with physician well-being from published literature [8-11,18-27]. Introducing this manual curation step, compared to relying solely on automated machine learning methods, aligns with prior literature demonstrating that incorporating clinician expertise into feature generation can improve model performance [28]. We also extracted physician gender and birth year (binned in 5-year increments) from the EHR database as well as practice type and clinic (both hashed to preserve confidentiality).

### Statistical Analysis

We used descriptive statistics including frequencies, means, and SDs to compare EHR use measures and demographics between survey responders and nonresponders using nonparametric methods to assess statistical significance, including Fisher exact test for categorical variables, Kruskal-Wallis test for continuous variables with unpaired observations, and Wilcoxon signed rank for continuous variables with paired observations. Reference values were set at the modal value (categorical variables) or the lowest value (ordinal variables).

Because the population of interest (nonresponders) may differ from the population available for model training (responders), we developed a weighting schema to tune the model for predictions more heavily weighted toward individuals less likely to respond. To develop these weights for our prediction model, we developed propensity scores quantifying the likelihood of survey response (vs nonresponse) in any given year based on EHR use measures. We used penalized logistic regression with 10-fold cross-validation to generate these propensity scores for survey response, using the full dataset of survey invitations, with the binary outcome of survey response defined as answering ≥4 out of 10 burnout prompts. We then used these propensity scores, denoted by *p*, to inform 3 approaches to weighting in our predictive models for burnout score, lending varying levels of higher weight to observations more similar to nonresponders (unweighted, 1/*p* weighting, and 1*−p* weighting). Unweighted predictions approximate the population of responders by ascribing equal weight to all observations in the training set. 1/*p*-Weighted predictions approximate the full population of responders and nonresponders by ascribing proportionately higher weight to responses with the lowest likelihood of response, with the lower limit of propensity scores truncated at 0.1 to prevent extreme weights. 1−*p*-Weighted predictions approximate the nonresponders by ascribing lower weight to responses with a higher likelihood of response.

To create our ensemble prediction model, we used a sample of always responders to train 3 separate prediction models, then used the remainder of always responders to calculate bias-correction coefficients governing the weight of each of the 3 models in the final ensemble model. To accomplish this task, we trained penalized linear regression models to predict burnout scores using a random 272 (80% sample) of the 340 always responders, grouped by physician. We generated 3 burnout predictions (1 for each weighting schema) for the remaining 68 (20% sample) of the 340 always responders. On this held-out test set, we then applied a bias-correction step in which we regressed actual burnout scores on the 3 predictions (1 from each weighting schema), then extracted the resultant β coefficients for each weighting schema as bias-correction coefficients, such that predictions consistently over- or underestimating burnout scores would be adjusted accordingly in the ensemble (bias-corrected) prediction.

Because nonresponders may represent a systematically different population than our training set of always responders, we chose to test predictive performance using the held-out test set of partial responders (physicians who responded to the survey in 1 year but declined to respond in the other year). We chose the partial responders as the test set because this group of physicians may most closely approximate nonresponders while still providing usable survey data in 1 year. Using the models generated in the training step and the coefficients generated in the bias-correction step, the performance of the ensemble model was tested to predict burnout scores for these partial responders. For ease of interpretation, we converted burnout predictions to the likelihood of burnout score ≥3.325 using a sigmoid distribution.

Because the partial responders may provide the most relevant data for predicting burnout among nonresponders, we then retrained the model using all responses (from always responders and the response years of partial responders) and used this final model to predict burnout scores among nonresponders. We combined these predicted burnout scores with the observed burnout scores from survey responses to estimate the population-level burnout burden.

Recognizing that we cannot directly assess the performance of our final predictions of nonresponders but that our sample contained a strong response rate, we also performed additional validation in which we synthetically reduced our response rate by pseudorandomly selecting a subset of physicians to be synthetic nonresponders. This approach allowed us to estimate applicability to institutions or years with lower response rates. For this synthetic survey response reduction, each person’s response propensity was jittered using a triangularly distributed random jitter from −0.5 to 0.5, centered at 0. Then we selected out the 20% of physicians with the lowest average response propensity as “candidate synthetic nonresponders.” We repeated this process 100 times, then chose any physician who was a candidate synthetic nonresponder in at least 50 cycles as a final synthetic nonresponder, to be added to the actual nonresponders. We retrained the model using only the limited set of responses, and then predicted burnout scores among the larger set of nonresponses.

Due to the hypothesis-generating nature of these analyses, no corrections were made for multiple testing. Statistical analyses were performed in Stata (version 17.0; StataCorp) and Python (version 3.3; Python Software Foundation) with modules scipy and sklearn.

## Results

**Figure 1.**
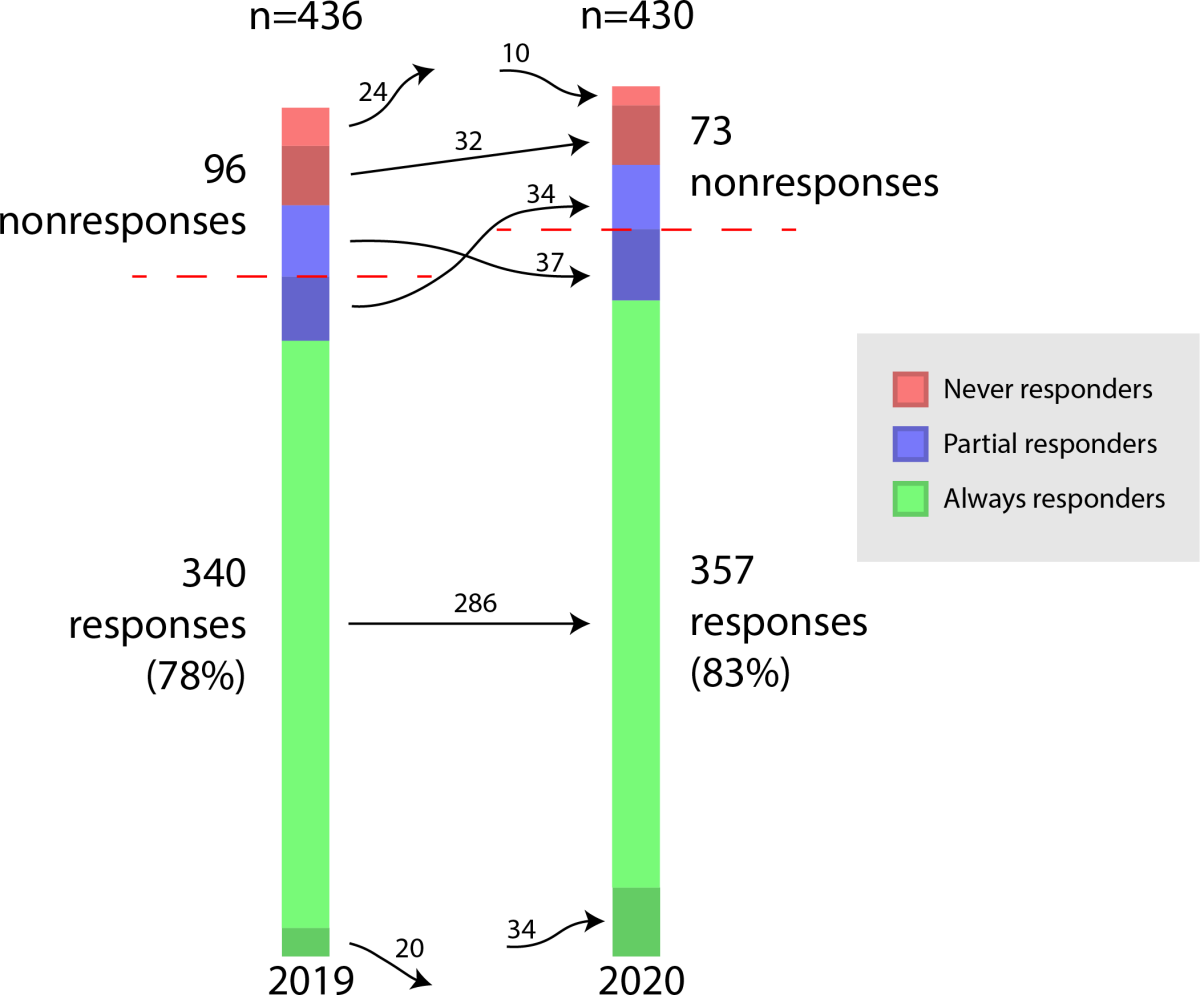
Response patterns of 477 primary care physicians administered a total of 866 surveys (1-2 surveys per physician) over a 2-year period.

A total of 477 unique physicians received 866 survey invitations (in 2019 alone: n=44, in 2020 alone: n=44, and in both years: n=389). The overall response rate was that 697 of 866 (80.5%) surveys were completed (in 2019: n=340, 78% and in 2020: n=357, 83%), with 66 never responders, 71 partial responders, and 340 always responders (Figure 1). Baseline demographics of the 3 groups of physicians are shown in Table 1.

**Table 1.** Baseline characteristics of never responders, partial responders, and always responders^a^.

	Responder group			*P* value^b^
	Always responders	Partial responders	Never responders
Physicians, n	340	71	66	—^c^
Surveys administered^d^, n	626	142	98	—
Surveys completed, n	626	71	0	—
**Demographics**				
Women, n (%)	204 (60)	53 (75)	38 (58)	.048
Age (years), median (IQR)	50 (40-60)	50 (40-60)	50 (35-60)	.88
**Workload and patient complexity measures, median (IQR)**				
Appointments	121.5 (58.5-184.0)	107.0 (45.0-161.0)	34.5 (0.0-115.0)	<.001
Appointments per work day	9.2 (6.2-11.7)	8.8 (6.9-11.7)	5.9 (0.0-9.6)	<.001
Laboratory orders	69.0 (15.0-173.5)	65.0 (11.0-156.0)	12.0 (0.0-71.0)	<.001
Mean number of patient conditions	167.9 (96.7-348.1)	275.9 (144.9-399.2)	206.8 (48.0-374.1)	.001
Number of messages	567.0 (277.0-953.5)	435.0 (86.0-891.0)	69.5 (0.0-279.0)	<.001
Number of notes written	187.0 (95.0-300.0)	149.0 (45.0-234.0)	33.0 (0.0-150.0)	<.001
Number of patients touched	212.0 (106.5-331.0)	148.0 (68.0-285.0)	48.5 (7.0-151.0)	<.001
**EHR^e^ efficiency measures, median (IQR)**				
Epic efficiency score	5.2 (4.0-6.2)	4.9 (3.6-5.8)	4.3 (0.0-5.6)	<.001
Minutes in Work Outside of Work per 8 hours of appointments	24.5 (11.5-47.6)	21.2 (4.5-47.2)	3.0 (0.0-20.7)	<.001
Minutes during pajama time	180.3 (21.3-599.5)	95.3 (22.7-389.9)	15.1 (0.0-132.3)	<.001
Minutes per off day	31.8 (13.5-66.0)	29.3 (5.1-59.1)	0.2 (0.0-19.0)	<.001
Minutes per 8 hours of appointments	173.1 (114.3-247.1)	189.6 (119.2-242.1)	120.8 (0.0-181.0)	<.001
Minutes in chart review per appointment	2.0 (1.1-3.7)	2.5 (1.3-3.7)	1.0 (0.0-3.8)	.003
Minutes in inbasket per 8 hours of appointments	31.7 (15.4-48.1)	29.1 (9.2-48.1)	9.6 (0.0-27.9)	<.001
Minutes in notes per 8 hours of appointments	60.9 (32.8-97.3)	60.2 (34.9-93.9)	42.8 (0.0-68.6)	<.001
Minutes in prescriptions per 8 hours of appointments	24.8 (14.5-38.2)	27.2 (14.5-36.4)	12.0 (0.0-30.4)	<.001
Average typos per note	1.2 (1.1-1.3)	1.1 (1.0-1.4)	1.0 (0.0-1.3)	<.001
Average words per note	396.2 (259.4-575.5)	402.6 (246.4-623.4)	264.5 (77.1-481.1)	<.001
Undivided attention (estimated)	0.7 (0.5-0.8)	0.6 (0.4-0.7)	0.5 (0.0-0.7)	.002
**EHR proficiency measures**				
Epic proficiency score, median (IQR)	7.0 (5.4-8.5)	6.5 (3.8-8.0)	3.1 (0.0-6.3)	<.001
Chart search, n (%)	325 (95.6)	64 (90)	46 (70)	<.001
Number of shared smartphrases, median (IQR)	140.0 (60.0-288.5)	184.0 (66.0-381.0)	40.5 (0.0-191.0)	<.001

a Data shown represent the month leading up to the most recent survey administration within the study period for each physician.

b *P* values obtained using the Fisher exact test (categorical variables) or the Kruskal-Wallis test (continuous variables).

c Not applicable.

d Physicians who joined or left the organization between survey administrations were only eligible to receive 1 survey. They were categorized with “always responders” if they responded or “never responders” if they did not respond. Full details are illustrated in Figure 1.

e EHR: electronic health record.

EHR use patterns for the 3 groups of physicians are also shown in Table 1, with never responders showing generally lower clinical workload and overall EHR proficiency, always responders showing generally higher clinical workload and EHR proficiency, and partial responders with intermediate values. In particular, never responders had a median of 5.9 (IQR 0.0-9.6) appointments per work day compared to 8.8 (IQR 6.9-11.7) for partial responders and 9.2 (IQR 6.2-11.7) for always responders. Although never responders exhibited the lowest summary EHR efficiency scores (partial responders: median 4.3, IQR 0.0-5.6 vs median 4.9, IQR 3.6-5.8 and always responders: median 5.2, IQR 4.0-6.2), they also showed the lowest minutes in notes per 8 hours of appointments (partial responders: median 42.8, IQR 0.0-68.6 vs median 60.2, IQR 34.9-93.9 and always responders: median 60.9, IQR 32.8-97.3), lowest minutes in EHR in basket per 8 hours of appointments (partial responders: median 9.6, IQR 0.0-27.9 vs median 29.1, IQR 9.2-48.1 and always responders: median 31.7, IQR 15.4-48.1), and lowest minutes in chart review per appointment (partial responders: median 1.0, IQR 0.0-3.8 vs median 2.5, IQR 1.3-37 and always responders: median 2.0, IQR 1.1-3.7).

Among the 71 partial responders, the year in which a response was given showed several differences in comparison to the nonresponse year as shown in Table 2. In particular, physicians responded in years in which they had higher clinical workload (eg, more appointments per work day: median 10.1, IQR 8.0-12.2 vs median 8.3, IQR 6.2-12.3; *P*=.03) but relatively similar EHR efficiency measures (eg, similar Epic efficiency score: median 5.0, IQR 4.1-5.9 vs median 4.7, IQR 3.4-5.8; *P*=.14). The results were similar when stratified by physicians who responded the first year and failed to respond the second year versus those with the reverse chronology.

**Table 2.** Electronic health record (EHR) use measures for the 71 partial responders, during nonresponse years and response years^a^.

	Nonresponse year	Response year	*P* value
Physicians, n	71	71	N/A^b^
**Workload and patient complexity measures, median (IQR)**			
Appointments	97.0 (41.5-151.0)	138.0 (65.0-192.0)	.001
Appointments per work day	8.3 (6.2-12.3)	10.1 (8.0-12.2)	.03
Laboratory orders	80.0 (2.0-139.0)	92.0 (14.0-203.0)	.001
Mean number of patient conditions	253.1 (131.1-408.6)	275.9 (125.6-375.7)	.51
Number of messages	309.0 (91.5-839.0)	462.0 (138.0-957.5)	.002
Number of notes written	145.0 (36.0-234.0)	184.0 (85.0-325.5)	.001
Number of patients touched	134.0 (57.0-250.0)	175.0 (82.5-313.0)	<.001
**EHR efficiency measures, median (IQR)**			
Epic efficiency score	4.7 (3.4-5.8)	5.0 (4.1-5.9)	.14
Minutes in work outside of work per 8 hours of appointments	17.7 (1.8-42.1)	22.3 (7.5-54.1)	.04
Minutes during pajama time	54.4 (0.0-321.7)	42.0 (0.0-201.0)	.51
Minutes per off day	32.3 (0.0-51.8)	34.6 (11.0-63.6)	.15
Minutes per 8 hours of appointments	184.3 (93.4-237.1)	183.8 (121.5-263.9)	.52
Minutes in chart review per appointment	2.4 (1.0-4.0)	2.3 (1.4-3.5)	.64
Minutes in inbasket per 8 hours of appointments	26.6 (8.6-48.3)	24.6 (11.0-45.4)	.71
Minutes in notes per 8 hours of appointments	59.6 (22.2-87.4)	60.2 (40.6-100.2)	.22
Minutes in prescriptions per 8 hours of appointments	25.5 (10.4-35.3)	28.0 (16.5-44.4)	.12
Average typos per note	1.1 (1.0-1.3)	1.1 (1.0-1.4)	.37
Average words per note	394.7 (257.2-593.7)	417.8 (274.4-625.4)	.61
Undivided attention (estimated)	0.6 (0.4-0.7)	0.7 (0.5-0.8)	.03
**EHR proficiency measures**			
Epic proficiency score, median (IQR)	5.8 (2.5-7.7)	6.4 (3.8-8.0)	.13
Chart search, n (%)	61 (86)	67 (94)	.16
Number of shared smartphrases, median (IQR)	122.0 (46.5-353.0)	225.0 (62.0-375.5)	.04

a *P* values obtained via Wilcoxon signed rank test.

b N/A: not applicable.

**Figure 2.**
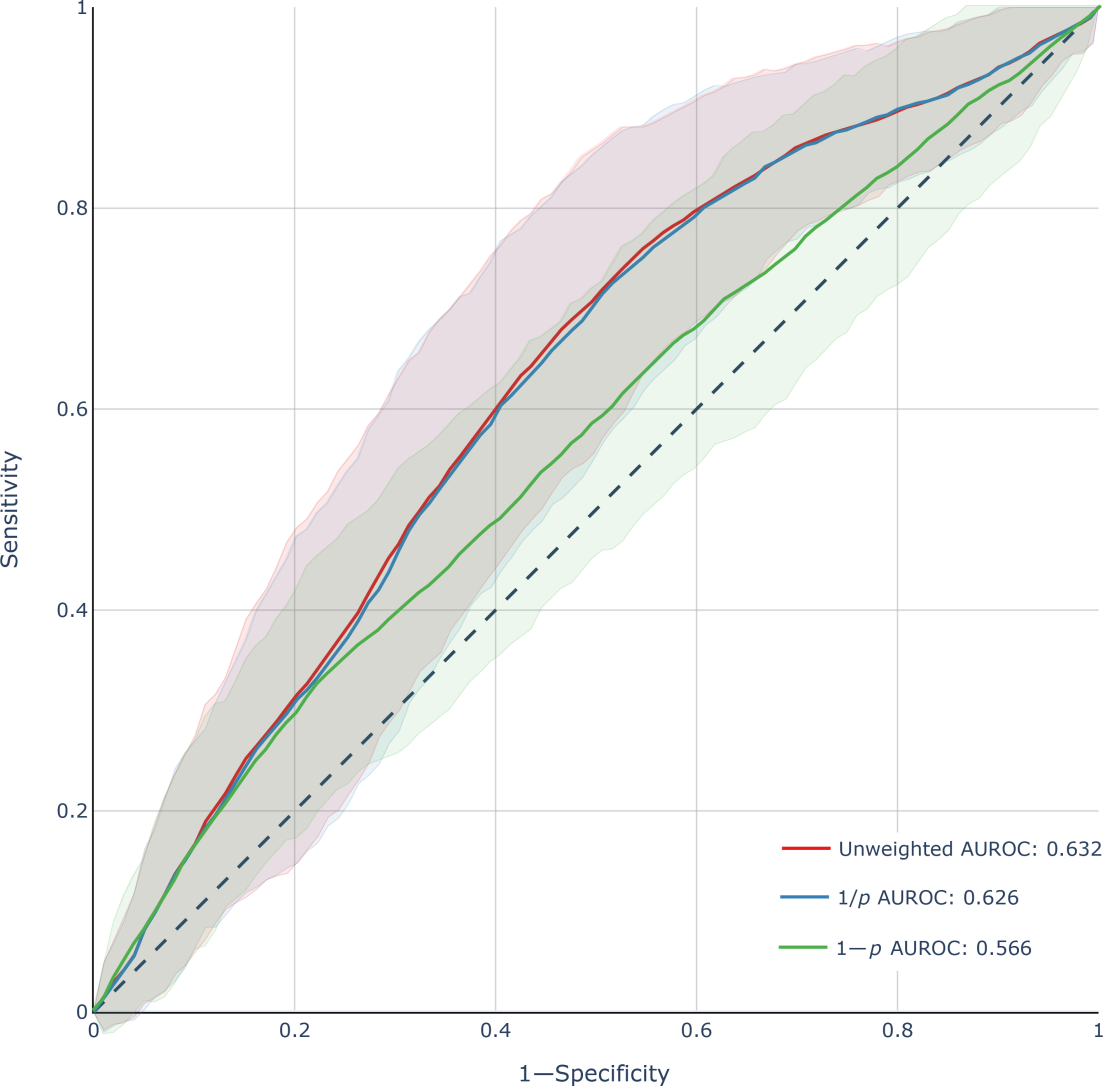
Receiver operating characteristics curves for 3 burnout prediction models: unweighted, weighted by inverse probability of survey response (1/p), and weighted by reversed probability of survey response (1*−p*). Results for the held-out test set of partial responders shown. Shaded areas represent 95% CIs obtained using bootstrap resampling methods. AUROC: area under the receiver operating characteristic curve.

**Figure 3.**
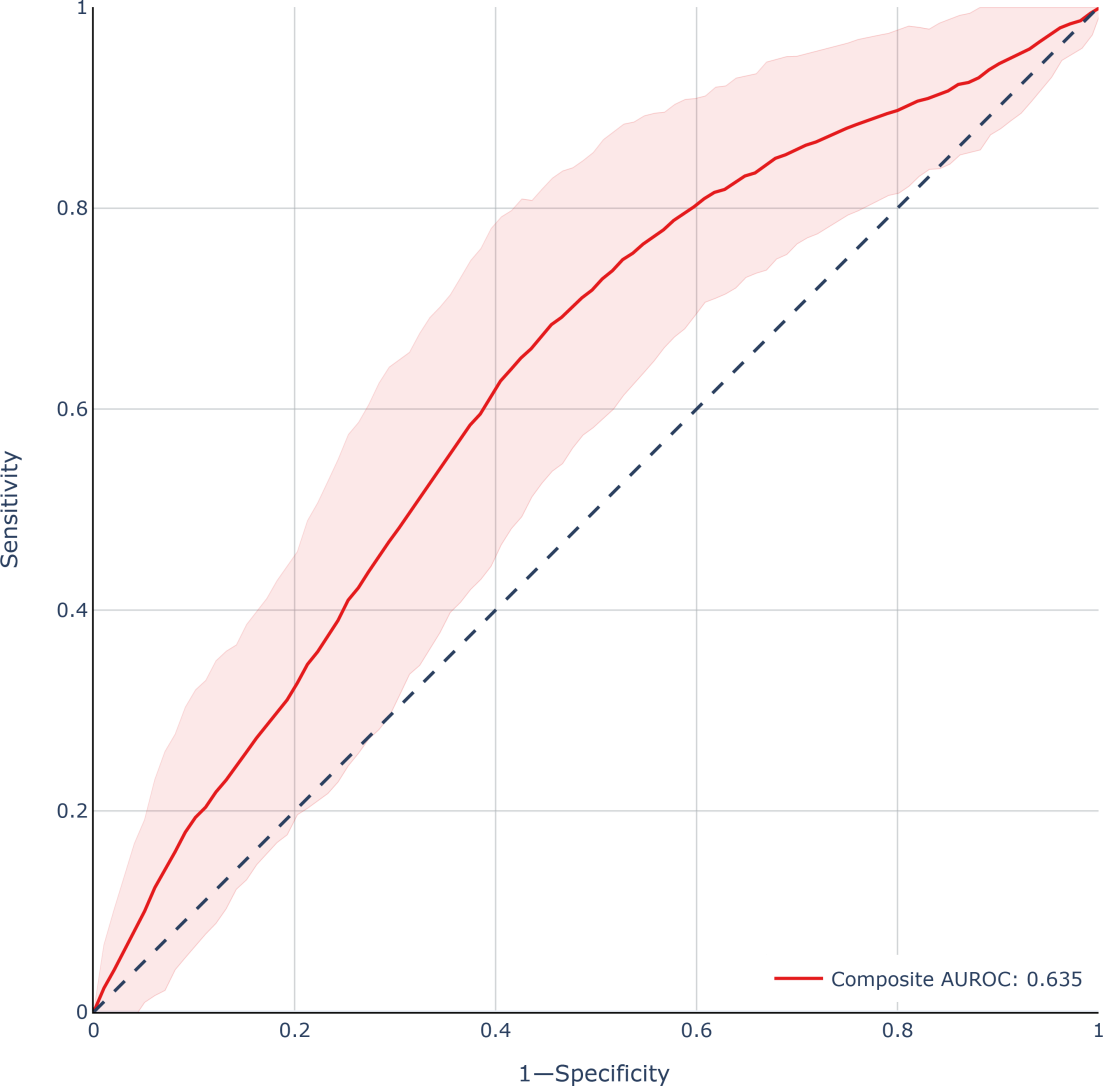
Receiver operating characteristics curve for the bias-corrected ensemble burnout prediction model. Results for the held-out test set of partial responders shown. Shaded area represents 95% CI obtained using bootstrap resampling methods. AUROC: area under the receiver operating characteristic curve.

Prediction models for survey response using multivariable logistic regression achieved an area under the receiver operating characteristic curve (AUROC) of 0.88 (95% CI 0.77‐0.91) as determined by 10-fold cross-validation grouped by physician. Prediction models for burnout score using multivariable linear regression with subsequent sigmoid transformation to probability of burnout achieved an out-of-sample AUROC of 0.63 (95% CI 0.57‐0.69) on the unweighted model, 0.62 (95% CI 0.57‐0.69) on the 1/*p* weighted model, and 0.57 (95% CI 0.50‐0.62) on the 1–p weighted model as shown in Figure 2. The ensemble model achieved an out-of-sample AUROC of 0.63 (95% CI 0.57‐0.70), sensitivity of 62%, specificity of 64%, and area under the precision-recall curve of 0.39 (95% CI 0.33‐0.48) for predicting burnout symptoms as shown in Figure 3.

After final training on all available data (see Multimedia Appendix 1 for coefficients from the top-performing model), the ensemble model predicted 52% (95% CI 41%‐63%) burnout prevalence among the nonresponders, higher than the 28% rate observed in responders. These predictions suggest a population-level estimated 31% (95% CI 30%‐33%) burnout prevalence, after incorporating the observed 28% burnout prevalence among responders.

In secondary analysis with synthetic response rate reduction, the observed burnout prevalence was 26% (excluding the synthetic nonresponders—individuals whose responses were concealed for the purposes of this secondary analysis), while the predicted burnout prevalence was 46% (including predictions for true nonresponders and synthetic nonresponders). The resultant population-level estimate of 33% burnout prevalence represented a 2% increase in estimated burnout compared to the fully informed model.

## Discussion

### Principal Findings

In this study, we found well-being survey responses to be more common among physicians with higher clinical workload and higher EHR proficiency measures, with a strong ability to predict survey response status using routinely collected EHR measures. However, we achieved lower predictive performance to predict burnout scores, which were better (AUROC 0.63, 95% CI 0.57-0.70) than chance (AUROC 0.5), but below the threshold for clinical actionability when predicting outcomes in an individual (typically AUROC of at least 0.8). The ability of EHR measures to predict burnout was not markedly improved by including propensity weighting schemas, though it remains possible that predictions on true nonresponders are more accurate as a result or that this approach may inform survey analysis strategies for other applications. Although we found predicted burnout scores among nonresponders to be higher than observed burnout scores among responders, there remains uncertainty regarding the magnitude of difference.

### Comparison to Prior Work

Previous studies looking for systematic differences between well-being survey responders and nonresponders have consistently failed to show meaningful differences. Using a large national sample of physicians, Shanafelt et al [29] reported no substantial differences between early responders and late responders nor between responders to the initial survey versus an incentivized, multimodality survey of nonresponders (2 standard approaches to evaluate for response bias) [1,4]. Ortega et al [30] reported no differences in burnout prevalence among responders to 3 consecutive surveys versus those who provided only 1 or 2 responses. Cardell et al [31] also reported no differences in burnout among responders who fully completed a survey and those with selective responses. These findings are in line with other studies of physician surveys in ambulatory practice or oncology care [32-34]. Studies that identified demographic differences by response status primarily note small differences in age, gender, specialty, and urbanicity [35-38].

Our study largely confirms these findings, with similar demographics among all responder groups, but extends these findings by evaluating highly granular EHR measures related to the work environment of physicians, providing much greater detail than has previously been available for nonresponders. Although we are unable to directly evaluate the predictive accuracy of our model on nonresponders, our findings suggest that in this sample of physicians, nonresponders have EHR use measures more similar to physicians who reported higher symptoms of burnout. Notably, these nonresponders tended to exhibit a lower volume of clinical workload, but many of the predictive features in our model related rather to the composition of the clinical workload (ie, the proportion of appointments that are level 5 rather than the raw number of appointments). However, the relatively weak predictive performance of this model to predict burnout (when evaluated against the surrogate group of partial responders) suggests caution in interpreting the magnitude of any such effect. In addition, the 1-year interval between the survey time points and response or nonresponse years of the partial responders may reflect important differences in burnout symptoms or work experiences, further distancing their relevance as a surrogate test population.

### Strengths and Limitations

Although burnout prediction accuracy falls below the threshold of actionability consistent with prior studies [14,15], the directionality of the difference between observed and predicted burnout may be valuable as a qualitative measure. Our findings suggest that institutions could leverage population-level EHR measures to identify if their nonresponders are expected to have better, worse, or similar symptoms to those measured among responders. In this way, comparisons may be better informed, particularly across years with different survey response rates. Although burnout was evaluated as a use case in this study, it remains possible that this approach may be more effective for predicting response to other survey instruments, particularly those with more direct conceptual connections with EHR use metadata (eg, EHR experience, control over schedule, or sleep-related impairment).

This study should be viewed in light of its design. As an observational study, we cannot infer any causality of the observed associations between EHR measures and burnout symptoms. Additionally, the nonresponders have survey results that are by definition not observable, and it remains possible that additional differences exist between nonresponders and our surrogate populations of partial responders and low-propensity responders. While this study population represents a large sample of academic and community primary care physicians, the results may not generalize to other specialties or institutions, and the necessary deidentification process prevented us from knowing the practice setting or clinic names of individual respondents. The response rates among this sample of physicians were relatively high due to strong institutional efforts to maximize responses, which may limit generalizability to institutions with lower response rates. The survey time points occurred both prior to and during the early phase of the COVID-19 response, which provided an exogenous shock to the health care delivery system including fluctuating visit volumes and a transition toward telehealth that may have unmasked relevant associations. However, changes in physician’s work and personal lives since that time may affect future results. Although we evaluated burnout prediction as a use case, this framework applied to other survey measures may provide better or worse predictive performance.

### Future Directions

We achieved moderate to strong performance in predicting survey response versus nonresponse. This finding highlights an opportunity to improve response rates by better targeting or incentivizing individuals predicted as less likely to respond. In general, the nonresponders appeared to exhibit a less clinically engaged phenotype with a lower workload and fewer EHR customizations. This phenotype may be reflective of reduced clinical load due to exhaustion, other academic or nonclinical responsibilities, vacation or sabbatical, or competing responsibilities in personal life. Decreased EHR proficiency as defined by EHR customizations may occur for many reasons, including lower clinical workload that may provide a lower payoff to an individual’s efforts toward customization, perceptions of helplessness or reduced motivation to engage in available tools, or unfamiliarity with available customizations.

Allowing physicians to self-select their survey delivery format or providing protected time for survey completion [30,39-42] may improve response rates. In particular, evaluating phenotypes associated with nonresponse may further inform survey timing, delivery modes, or incentive structures to maximize response rates. For example, an institution that predicates a year-end clinical bonus on survey completion may inadvertently oversample those with high clinical loads, as their incentive will carry relatively more value. Alternatively, an institution that provides survey invitations and reminders during daytime hours or posted only in clinical locations may inadvertently reduce engagement by physicians with infrequent or off-hours clinical work.

### Conclusions

In this sample of primary care physicians in a large academic medical center and its community affiliates, we found that differences in EHR use measures may enable the prediction of physician survey response with moderate to strong accuracy, but that EHR use measures have limited ability to predict burnout symptoms. Our finding that nonresponders had EHR use measures more similar to responders with symptoms of burnout suggested that, if anything, nonresponders may have higher rates of burnout than responders. Further studies are needed to validate these findings in other settings and over a longer time horizon.

## Supplementary material

10.2196/64722 Multimedia Appendix 1 Standardized coefficients from top-performing model for features selected via penalized linear regression. Features are ordered by coefficient value, with the highest listed features associating with higher burnout scores and the lowest listed features associating with lower burnout scores.

## Abbreviations

AUROC: area under the receiver operating characteristic curve
EHR: electronic health record
