# Supplementary material for: Electronic Health Record Use Patterns Among Well-Being Survey Responders and Nonresponders: Longitudinal Observational Study
Source: JMIR Med Inform. 2025 Feb 4;13:e64722. doi: 10.2196/64722 (PMC11813195; doi:10.2196/64722)
Supplement: Multimedia Appendix 1 [file medinform-v13-e64722-s001.docx]

| **Feature** | **Standardized Coefficient** |
| --- | --- |
| Minutes in chart review per day | 0.116 |
| Number of messages from team members per day | 0.091 |
| Proportion of appointments that are Level 5 established | 0.069 |
| Proportion of appointments that are Level 5 consults | 0.046 |
| Proportion of lab orders using system preferences | 0.032 |
| Number of shared SmartPhrases | 0.023 |
| Number of Quick Filters owned by the physician | 0.021 |
| Increase in number of QuickActions available | 0.014 |
| Proportion of imaging orders using system preferences | 0.008 |
| Minutes spent finishing incomplete notes | 0.000 |
|  |  |
| Number of repeat patients per month | -0.010 |
| Proportion of radiology orders using user preferences | -0.012 |
| Increase in number of procedure notes per month | -0.016 |
| Proportion of note characters that are self-written | -0.016 |
| 3-month average of proportion of note characters that are self-written | -0.019 |
| Male (vs. female) | -0.026 |
| Age 60-65 | -0.031 |
| Number of items in the user preference list | -0.034 |
| 3-month average of proportion of appointments that are Level 3 new | -0.052 |
| Age >= 65 | -0.067 |
| Proportion of appointments that are Level 3 established | -0.080 |
| 3-month average of patient age | -0.085 |
| Number of patient deaths per month | -0.095 |
| Proportion of appointments that are Level 4 new | -0.098 |
